# Adrenaline-resistant anaphylactic shock caused by contrast medium in a patient after risperidone overdose: a case report

**DOI:** 10.1186/s40780-023-00292-z

**Published:** 2023-07-12

**Authors:** Takafumi Nakano, Yoshihiko Nakamura, Keisuke Sato, Yoshito Izutani, Hiroto Iyota, Misaki Aoyagi, Taisuke Kitamura, Toshinobu Hayashi, Koichi Matsuo, Kenichi Mishima, Hidetoshi Kamimura, Hiroyasu Ishikura, Takashi Egawa

**Affiliations:** 1 Department of Pharmacy, Fukuoka University Hospital, 7-45-1 Nanakuma, Jonan-ku, Fukuoka, 814-0180 Japan; 2 Department of Pharmaceutical and Healthcare Management, Faculty of Pharmaceutical Sciences, Fukuoka University, 8-19-1 Nanakuma, Jonan-ku, Fukuoka, 814-0180 Japan; 3 Department of Emergency and Critical Care Medicine, Fukuoka University Hospital, Fukuoka, Japan; 4 Department of Physiology and Pharmacology, Faculty of Pharmaceutical Sciences, Fukuoka University, Fukuoka, Japan

**Keywords:** Risperidone, Overdose, Anaphylactic shock, Contrast media

## Abstract

**Background:**

In Japan, the use of risperidone in combination with adrenaline is contraindicated, except in cases of anaphylaxis. Therefore, there is limited clinical evidence regarding the interaction of these two drugs. Here, we report the clinical course of a case of adrenaline-resistant anaphylactic shock induced by a contrast medium injection after a risperidone overdose.

**Case presentation:**

A man in his 30s was transported to our hospital after attempting suicide by taking 10 mg of risperidone and jumping from a height of 10 m. To determine the location and severity of his injuries, he was injected with an iodinated contrast medium, after which he developed generalized erythema and hypotension and was diagnosed with anaphylactic shock. A 0.5 mg dose of adrenaline was administered with no improvement, followed by another 0.5 mg dose that did not change his blood pressure. After infusion of a sodium bicarbonate solution (8.4%), administration of fresh frozen plasma, and additional administration of adrenaline (0.6–1.2 µg/min), his blood pressure improved, and he recovered from the anaphylactic shock.

**Conclusions:**

This was a rare case of a risperidone overdose followed by adrenaline-resistant anaphylactic shock. The resistance is likely associated with the high blood concentration of risperidone. Our findings indicate that the potential for decreased adrenergic responsiveness should be considered in patients undergoing risperidone treatment in the event of anaphylactic shock.

## Introduction

In Japan, the use of antipsychotic drugs with α-blocking activity in combination with adrenaline is not recommended; the concomitant administration of α-blockers and adrenaline can increase the risk of hypotension, as the β-agonistic effects of adrenaline will prove dominant [1]. However, adrenaline is the first-line medication for anaphylaxis, so this contraindication may be problematic in clinical practice if a patient with schizophrenia who receives antipsychotic drugs also develops anaphylaxis. A survey by the Pharmaceutical and Medical Device Agency (PMDA) revealed only a few cases of hypotension in patients co-administered with adrenaline and antipsychotics with α-blocking activity; all of these had a favorable outcome (i.e., recovery) [2]. Moreover, in the United States and Europe, the package insert for adrenaline does not state that concomitant use with α-blocking antipsychotics is contraindicated [3]. The rationale for this contraindication is based on theoretical pharmacological effects rather than on accumulated evidence from clinical reports and case studies [4]. Therefore, in 2018, the contraindication of the use of adrenaline with α-blocking antipsychotics, such as risperidone, was removed specifically for the treatment of anaphylaxis. However, there are few reports on anaphylaxis in patients treated with α-blocking antipsychotics. Considering that adrenergic responsiveness may vary with the dosage of antipsychotics, it is important to accumulate clinical evidence on the concomitant use of antipsychotics and adrenaline.

Here, we report a case of adrenaline-resistant anaphylactic shock induced by a radiographic contrast medium in a patient who took a 10 mg dose of risperidone. The common maintenance dosage of risperidone is 2–6 mg/day, and the daily dose should not exceed 12 mg. Furthermore, if a patient is required to take 12 mg of risperidone, it should be divided into two doses per day. Page et al. also defined risperidone overdose as > 6 mg per dose [5]. Therefore, we considered that 10 mg of risperidone was a clinically problematic dose, and defined this case as that of a risperidone overdose.

## Case

The patient, a man in his 30s, had a history of schizophrenia, pollinosis, and adverse reactions to unspecified drugs, such as gastrointestinal medications. He was receiving regular treatment for schizophrenia, which included antipsychotics and sleeping pills (Table 1).

**Table 1:** Medication history of the patient

Name of the drug	mg/day	(/a day)
Risperidone	6.0	Three times
Brexpiprazole	2.0	Twice
Guanfacine hydrochloride	4.0	Once
Biperiden hydrochloride	3.0	Three times
Lorazepam	3.0	Three times
Clonazepam	1.5	Three times
Topiramate	300.0	Three times
Ramelteon	8.0	Once
Ethyl loflazepate	2.0	Once
Suvorexant	20.0	Once

The patient attempted suicide by taking 10 mg of risperidone and jumping from a height of 10 m; after this, he was urgently transported to our hospital. On admission, the patient’s level of consciousness was I-1 on the Japan Coma Scale and 14 (E3-V5-M6) on the Glasgow Coma Scale. His vital signs were as follows: blood pressure, 70/48 mm/Hg; respiratory rate, 24 breaths/min; and pulse rate, 79 beats/min. Focused assessment with sonography in trauma (FAST) examination was performed in the department of emergency and found to be negative. However, the patient had suffered a partially unstable pelvic fracture, including a right pubic bone fracture, coccygeal fracture, and sacrum fracture. The patient had also suffered an open fracture in the right arm, a lumbar fracture at L2, a left calcaneus fracture, and bilateral tibial plateau fractures. There was no active bleeding on the surface of his body. The patient’s laboratory values at admission are shown in Table 2. The patient had elevated hepatic enzymes, possibly due to injury or medication, and a low red blood cell count and hemoglobin level due to blood loss. Urine drug screening using SIGNIFY® ER (Abbott Japan LLC, Tokyo) was weakly positive for benzodiazepines only. However, we heard from his family that no shells of any drugs (other than risperidone) were found. Furthermore, the patient did not exhibit a coma that would have been likely in the event of a benzodiazepines overdose. Therefore, we considered that the positive of benzodiazepines was due to daily drug treatment. Alcohol ingestion was also ruled out based on the osmolal gap. Based on these findings, the patient was diagnosed with an overdose of risperidone.

**Table 2:** Results of the blood test performed on hospital arrival

[Blood count / biological]		[Blood gas / coagulation and fibrinolytic system]	
WBC	6.5×10^3^/µL	pH	7.362
RBC	430.0×10^4^/µL	PCO_2_	30.0 mmHg
Hemoglobin	13.1 g/dL	PO_2_	117.0 mmHg
Platelet	19.4×10^4^/µL	HCO_3_^−^	16.6 mmol/L
Albumin	4.2 g/dL	BE	-7.2 mmol/L
CRP	0.02 mg/dL		
BUN	7.0 mg/dL	PT-INR	1
Cr	1.2 mg/dL	APTT	27.9S
Na	135 mmol/L	FDP	82.0 µg/mL
K	3.4 mmol/L	D-dimer	45.9 µg/mL
Cl	105.0 mmol/L		
Total bilirubin	0.6 mg/dL	Osmolal Gap	0 mOsm/kg
AST	117.0 IU/L		
ALT	105.0 IU/L	[Score]	
ChE	358.0 IU/L	APACHE-II	9 Points
LDH	549.0 IU/L	SOFA	5 Points
CK	270.0 IU/L	ISS	22 Points
Glucose	112.0 mg/dL		
Lactic acid	27.6 mg/dL		

ALT, alanine aminotransferase; APACHE, acute physiology and chronic health evaluation; APTT, activated partial thromboplastin time; AST, aspartate aminotransferase; BE, base excess; BUN, blood urea nitrogen; ChE, cholinesterase; Cr, creatinine; CK, creatine kinase; CRP, C-reactive protein; FDP, fibrin degradation product; ISS, injury severity score; LDH, lactate dehydrogenase; PT-INR, prothrombin time-international normalized ratio; RBC, red blood cell; SOFA, sequential organ failure assessment; WBC, white blood cell

**Fig. 1:**
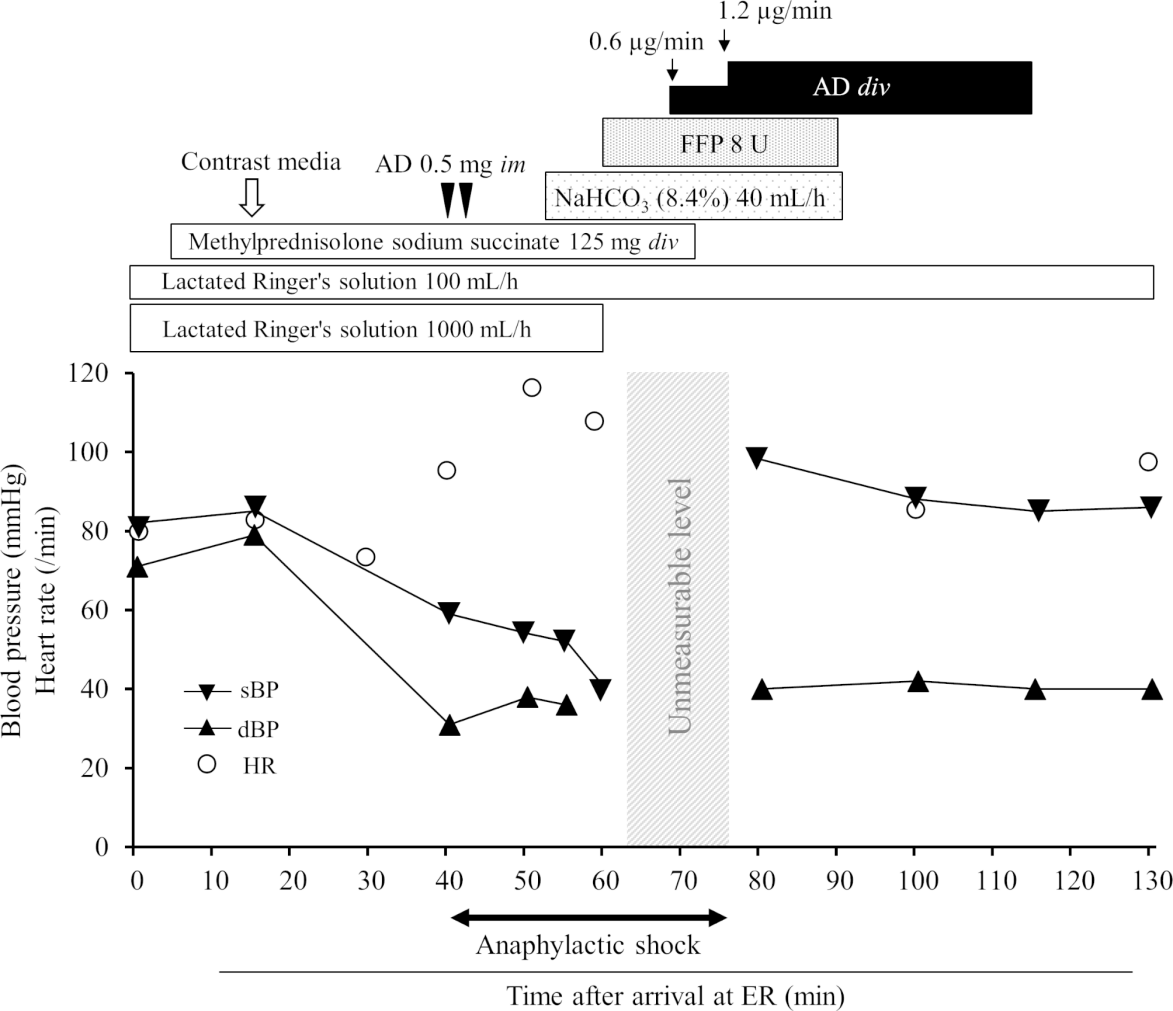
Clinical course of the patient AD, adrenaline; dBP, diastolic blood pressure; div; drip infusion into vein; ER, emergency room; FFP, fresh frozen plasma; HR, heart rate; im, intramuscular injection; sBP, systolic blood pressure

The patient’s clinical course since admission is shown in Fig. 1. An iodinated contrast medium was prepared for detailed examination of the injured areas. Due to his history of unspecified drug allergies, 125 mg methylprednisolone sodium succinate was administered prior to contrast medium administration, and contrast-enhanced CT was performed. Following administration of the contrast medium, the patient developed generalized erythema, wheezing, and hypotension, subsequently resulting in a diagnosis of an anaphylactic shock. An intramuscular injection of 0.5 mg adrenaline was administered, which was then followed by a second adrenaline injection of an identical dose and route when no improvement of hypotension was observed; however, his blood pressure remained unchanged. Therefore, considering the risk of complications from metabolic acidosis and hemorrhagic shock, 8.4% sodium bicarbonate solution (40 mL/h) and 8 units of fresh frozen plasma were administered. Furthermore, the administration mode of adrenaline was switched to a continuous intravenous infusion (0.6–1.2 µg/min). Subsequently, his blood pressure improved, and it did not drop even after the continuous intravenous infusion of adrenaline was discontinued, indicating that the patient had recovered from the anaphylactic shock.

The plasma concentration of risperidone in the patient’s blood samples collected at admission was revealed to be 25.8 ng/mL by liquid chromatography–tandem mass spectrometry (ACQUITY Ultra Performance LC and Quattro Premier XE; Waters Corporation, Japan).

After the patient regained full consciousness, we confirmed that he had not taken any medications other than risperidone before jumping.

Written consent for the publication of this case report was obtained from the patient in accordance with the Guidelines for Privacy Protection in Medical Papers and Conference Presentations, including Case Reports (Japan Surgical Society) [6]. This case report was not subject to a formal review by the Ethical Review Board.

## Discussion and conclusion

This was a rare case of adrenaline-resistant anaphylactic shock induced by a contrast medium following an overdose of risperidone. The clinical course of this case showed that risperidone may have a dose-dependent effect on adrenergic responsiveness. Because adrenaline is an important rescue drug for the treatment of anaphylaxis [7], clinical evidence of the concomitant use of adrenaline and antipsychotics with α-blocking activity should be accumulated.

Several etiological mechanisms of anaphylaxis have been described, but most episodes are triggered through an immunological mechanism involving IgE [8]. However, contrast media-induced anaphylaxis, previously classified as an anaphylactoid reaction, is usually IgE-independent [9, 10] and involves the release of histamine, activation of the complement system, and inhibition of enzymatic activity by the direct stimulation of mast cells and basophils. In rare cases, contrast media-induced anaphylaxis may be mediated by specific IgE antibodies and manifest as a Type I allergic reaction [9, 10]. The PMDA reported that diagnostic agents, including contrast media, induced 3428 cases of anaphylaxis between 2005 and 2017; these account for 20.3% of all anaphylaxis cases [11]. Within these cases, diagnostic agents were responsible for 120 fatalities (28.7% of all fatalities due to anaphylaxis) [11]. Anaphylaxis induced by contrast media during clinical examinations at medical institutions can be treated more rapidly than anaphylaxis associated with food or oral drugs; however, diagnostic agents were responsible for over 20% of all anaphylactic deaths. Therefore, contrast agent-induced anaphylaxis is a pathological condition that requires the attention of researchers and clinicians alike.

Because the patient had a history of unspecified drug-induced allergies, he was premedicated with steroids prior to the administration of the contrast medium in an attempt to prevent the onset of allergic symptoms. The American College of Radiology recommends that premedication be performed at least several hours or even a day prior to contrast media administration for best results [12]. However, as the patient was urgently hospitalized, the steroid was administered just prior to the administration of the contrast medium, which may have reduced its prophylactic efficacy.

In the treatment of anaphylaxis, adrenaline is usually administered once or twice at a dose of 0.5 mg via intramuscular injection, and this has been reported to be effective for the Japanese physique [11]. However, although the patient received two doses of 0.5 mg adrenaline, his hypotension did not improve. Several factors may have contributed to the patient’s adrenergic resistance. The first was his body constitution. The patient’s body (height: 174 cm; weight: 82.4 kg) was slightly larger than that of the average Japanese adult man. However, his body build was not notably different from that of people in the Western countries. Thus, the effect of body size was considered minimal. Second, the pharmacological action of risperidone may have antagonized the α-stimulating activity of adrenaline due to its high plasma concentration. Dopamine D2 and 5-HT2 antagonists, such as risperidone, possess antipsychotic properties [1]. Furthermore, laboratory animal studies have shown that risperidone exerts strong α_1_ and mild α_2_ receptor-blocking effects [1]. Therefore, in Japan, the concomitant use of antipsychotic drugs with α-blocking activity (such as risperidone and aripiprazole) and adrenaline was contraindicated because the β-agonistic effect of adrenaline would dominate and increase the risk of hypotension. However, in 2018, this contraindication was removed only for the treatment of anaphylaxis, because adrenaline is essential in anaphylaxis treatment. In fact, there are few reports that adrenaline caused severe hypotension due to suspected adrenaline reversal by the concomitant use of adrenaline and antipsychotics except for chlorpromazine [3, 13]. Therefore, adrenaline was the best option in this case as well, even though the patient was treated with risperidone. The fact that a continuous intravenous infusion of adrenaline was required to improve the patient’s blood pressure may be due to the risperidone overdose. Risperidone and adrenaline are competitive antagonists of α_1_ receptors; thus, their relative effects are dependent on the drug concentration. In a Japanese male adult, the C_max_, T_max_, and T_1/2_ of risperidone after a dose of 2 mg are 13.6–16.0 ng/mL, 0.9–1.7 h, and 3.1–3.4 h, respectively [14, 15]. In this case, the time elapsed between ingestion and hospitalization was estimated to be 1–2 h. As this is closer to the T_max_ range than the T_1/2_ range reported above, we inferred that the blood concentration of risperidone in this patient would also be close to the peak value after ingestion. As the concentration of risperidone measured in the patient was over twice the C_max_ values obtained after ingestion of the standard 2 mg dose, we believe that the patient required multiple doses and a continuous intravenous infusion of adrenaline because of the high concentration of risperidone in his body. Meanwhile, complications such as metabolic acidosis and hemorrhagic shock may also have contributed to adrenergic resistance. A high H^+^ concentration in the blood, acidemia, affects the exchange of H^+^/Ca^2+^ and attenuates muscular contraction [16]. However, in this patient, the arterial blood pH was 7.36, indicating mild acidemia. Therefore, it was considered that acidemia did not contribute to adrenaline resistance. Because the patient also experienced bleeding due to pelvic fractures and other injuries, the abnormal body hemodynamics caused by blood loss may have prolonged his state of shock. Thus, the patient was administered with fresh frozen plasma considering the complications of a hemorrhagic shock prior to the initiation of the continuous intravenous infusion of adrenaline. Meanwhile, the patient did not present with tachycardia, which is often observed during a hemorrhagic shock, except for transient increase due to administration of adrenaline. Moreover, FAST findings were negative, no evidence of extravasation or retroperitoneal hematoma formation was observed, and his pelvic fracture did not require transcatheter arterial embolization. Based on these findings, the adrenaline-resistant nature of his anaphylactic episode was considered to be more likely due to risperidone overdose than hemorrhagic shock. However, based on the patient’s clinical course alone, we were unable to demonstrate that the high blood concentration of risperidone was associated with the adrenaline resistance observed during treatment of the anaphylaxis, and no other potential explanation could be identified. Further investigation of this phenomenon will require additional case reports and clinical data related to the concomitant use of adrenaline and risperidone.

Adrenaline is critical for the treatment of anaphylaxis due to its ability to increase the blood pressure and cardiac contractility by stimulating α_1_ and β_1_ receptors; it promotes bronchodilation by β_2_ receptor stimulation. Therefore, the role of adrenaline in the treatment of anaphylaxis will continue to be of utmost importance in the future, and its use should be recommended without hesitation. However, our case showed that the effects of adrenaline may be muted in patients receiving treatment with α-blocking antipsychotics such as risperidone; the dosage and time elapsed since administration may change the required minimum effective dose of adrenaline. In our case, the patient did not show hypotension induced by a dominance of the β2 agonistic effects by adrenaline reversal. However, if the blood concentrations of α-blocking drugs are higher, the adrenaline requirement would also increase; therefore, the reaction to adrenaline administration requires special attention.

## Data Availability

Not applicable.

## Abbreviations

FAST: focused assessment with sonography in trauma
PMDA: Pharmaceutical and Medical Device Agency
